# Noncirrhotic Extrahepatic Portosystemic Shunt Causing Adult-Onset Encephalopathy Treated with Endovascular Closure

**DOI:** 10.1155/2015/852853

**Published:** 2015-06-25

**Authors:** Eldad Elnekave, Eugenia Belenky, Lindsley Van der Veer

**Affiliations:** ^1^Department of Radiology and Oncology, Rabin Medical Center, 49100 Petah Tikva, Israel; ^2^Department of Radiology, Rabin Medical Center, 49100 Petah Tikva, Israel; ^3^Department of Gastroenterology, Easton Hospital, 250 S 21st Street, Easton, PA 18042, USA

## Abstract

A 54-year-old woman presented with a six-month history of episodic confusion and progressive ataxia. A comprehensive metabolic panel was notable for elevated values of alkaline phosphatase (161 U/L), total bilirubin (1.5 mg/dL), and serum ammonia of 300 umol/L (normal range 9–47). Hepatitis panel, relevant serological tests, tumor markers (CA-19-9, CEA), and urea cycle enzyme studies were unrevealing. Lactulose and rifaximin therapy failed to normalize serum ammonia levels. Imaging revealed a structural vascular abnormality communicating between an enlarged inferior mesenteric vein and the left renal vein, measuring 16 mm in greatest diameter. The diagnosis of congenital extrahepatic portosystemic shunt was made and endovascular shunt closure was performed using a 22 mm Amplatzer II vascular plug. Within a day, serum ammonia levels normalized. Lactulose and rifaximin were discontinued, and confusion and ataxia resolved.

## 1. Case Presentation

A 54-year-old female presented with a six-month history of episodic confusion and progressive ataxia. Her past medical history was significant for asthma and pancreatic adenocarcinoma for which she had undergone Whipple's procedure, chemotherapy, and external beam radiation five years earlier. At the time of presentation she had no evidence of residual disease. The patient reported a remote history of recreational drug use and social alcohol intake. A comprehensive metabolic panel was notable for elevated values of alkaline phosphatase (161 U/L) and total bilirubin (1.5 mg/dL) with a normal direct bilirubin component (0.3 mg/dL). Further laboratory evaluation was unremarkable except for serum ammonia of 300 umol/L. Hepatitis panel, relevant serological tests, tumor markers (CA-19-9, CEA), and urea cycle enzyme studies were unrevealing.

**Figure 1:**
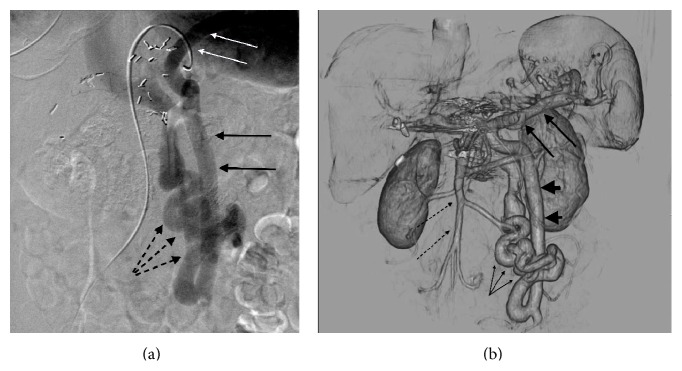
(a) Visceral phase angiography following injection of the splenic artery before occlusion of the portosystemic shunt shows retrograde flow from the splenic vein (white arrows) via an enlarged inferior mesenteric vein (black arrows) via shunt (three dashed arrows) into the systemic venous system. (b) Venous phase volume-rendered (VR) image demonstrates a serpentine vascular shunt (three small arrows) connecting an enlarged IMV (large arrow heads) to the left renal vein via a tortuous shunt (three small arrows). The splenic vein is marked with three two large arrows. Normal appearance of the SMV (dashed arrows). Streak artifact in the portal confluence is due to clips from prior Whipple procedure.

A computed tomography (CT) scan of the abdomen demonstrated a smooth hepatic contour and was without splenomegaly, perigastric varices, splenorenal varices, or ascites or other stigmata of portal hypertension. The extrahepatic portal vein was normal in caliber and contrast opacification. A serpentine vascular structure in the right lower abdominal quadrant communicated between a markedly enlarged inferior mesenteric vein (IMV) and the left renal vein (Figure 1). The vascular structure could be identified retrospectively on CT scans dating at least ten years earlier. The shunt progressively increased in size from a maximal diameter of 7 mm in 2003 to 16 mm in 2013. Doppler evaluation demonstrated low flow in the portal vein with a Time Averaged Mean Velocity (TAMV) of 10 cm/s (normal 15–18 cm/s). Transjugular liver biopsy showed fatty change and mild chronic portal triad inflammation and normal appearance of the portal vein within the triad. There was no pathologic evidence of cirrhosis and the corrected mean portosystemic venous gradient measured 3 mmHg.

Within two-month initiation of lactulose and rifaximin therapy, the patient was admitted with worsening encephalopathy. An interventional radiology consultation was requested and diagnostic angiography was performed. A large portal-systemic venous shunt was identified on venous phase of splenic arteriography, which showed sequential retrograde opacification of an enlarged inferior mesenteric vein (IMV), a serpentine shunt, and left renal vein. Flow in the portal vein was undetected (Figure 1). Interrogation of the inferior mesenteric artery (IMA) also revealed left colic and sigmoidal veins draining retrograde across the IMV to the shunt vessel. Superior mesenteric artery (SMA) angiography showed normal physiologic opacification of the superior mesenteric vein (SMV) with hepatopetal portal venous flow. Percutaneous transhepatic portography demonstrated normal caliber of the extrahepatic and central intrahepatic portal venous system with no focal stenosis or obstructing intraluminal lesion.

*Treatment Approach*. Endovascular shunt closure was found to represent the least invasive therapeutic option with a high probability of technical and clinical success. A systemic venous approach was preferred in consideration of the extreme tortuosity of the shunt vessel, which would preclude embolization near the systemic venous confluence. Following clinical consultation and discussion with the patient and her daughters, informed consent for intervention was obtained. The shunt was accessed with a 5-French Cobra-2 catheter (AngioDynamics Inc., Queensbury, NY) from the right femoral vein via the left renal vein. It measured 16 mm in diameter near the confluence with the left renal vein. The confluence of left ovarian vein to the renal vein was identified approximately 1 cm lateral to the shunt. A 22 mm Amplatzer II vascular plug (St. Jude Medical, Inc., St. Paul, Minnesota) was advanced within a 7 Fr sheath and deployed across 3 cm of the shunt near the confluence with the renal vein. The specifications were adherent to the manufacturer's recommendations to upsize the vascular plug by 30–50% of vessel diameter.

**Figure 2:**
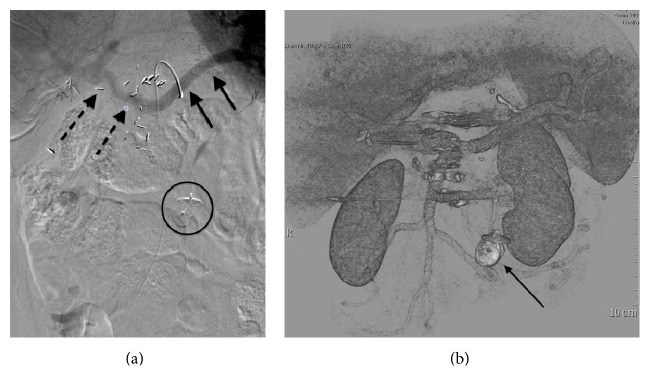
(a) Visceral phase angiography following injection of the splenic artery after occlusion of the portosystemic shunt with Amplatzer vascular plug (circled) demonstrates splenic vein (white arrows) draining antegrade into the portal vein (black arrows). (b) VR CT image shows the position of the Amplatzer plug (arrow) and successful embolization of the shunt (no longer visualized). Portal venous flow is now seen to and within the liver.

Following embolization, antegrade hepatopetal flow was documented in the splenic and inferior mesenteric veins (Figure 2). In anticipation of thrombogenic slow antegrade flow within the hypertrophied inferior mesenteric vein (which had served as a conduit for retrograde splenic outflow), prophylactic anticoagulation was initiated using low molecular weight heparin overlapping with warfarin continued as outpatient therapy (target INR 2.0–3.0). Follow-up imaging demonstrated partial thrombus in the hypertrophied inferior mesenteric vein without extension into the portal vein. There was no evidence of submucosal colonic edema or ascites.

The patient's serum ammonia levels normalized within twenty-four hours of the procedure. Lactulose and rifaximin were discontinued over 48 hours. The patient and her family reported immediate improvement in cognitive function and progressively improved gait. Follow-up CT scan performed one month later (Figure 2) showed successful shunt embolization. Nonocclusive thrombus was seen in the IMV, but not in the portal vein. Doppler evaluation of the main intrahepatic portal vein showed improved flow with a mean velocity of 16 cm/s. At 24-month follow-up the patient remains symptom-free.

## 2. Discussion

Spontaneous portosystemic shunts occur frequently in the setting of cirrhosis. Congenital portosystemic shunts are much less common: 316 cases had been reported as of 2013 [1], with the vast majority appearing in the pediatric literature. Congenital shunts are usually detected sonographically in utero or among infants who screen positive for galactosemia but negative for enzymatic deficiency [2].

The majority of pediatric shunts are intrahepatic, involving one or more communications between the portal vein and the hepatic veins or intrahepatic inferior vena cava (IVC). Clinically significant intrahepatic shunts are usually amenable to endovascular coil embolization [2] whereas small, clinically silent shunts may resolve spontaneously.

The anatomic classification [3] of congenital extrahepatic portosystemic shunts hinges on whether the intrahepatic portal vein is absent (type 1) or present (type 2). In type 1 congenital extrahepatic portosystemic shunt (CEPS), also known as Abernethy malformation, the portal vein is congenitally absent. In such cases the shunt represents the only mesenteric and splenic outflow and therefore liver transplant is the only therapeutic option [4]. Type 1 shunts typically present early in childhood [5] and are often accompanied by cardiac and/or renal anomalies.

In type 2 CEPS, the intrahepatic portal vein is normal but mesenteric and/or splenic flow is diverted away from it via a vascular anomaly. Type 2 CEPS may be diagnosed in infancy or adulthood [6].

Among noncirrhotic adults, the diagnosis of a symptomatic portosystemic shunt is exceedingly rare. Although altered vascular kinetics may result from malignant or benign portal venous strictures following laparotomy or external beam radiation, our patient's shunt predated surgical intervention by at least five years and no stricture was demonstrated during portal venography. Furthermore there was no direct or indirect evidence of portal hypertension. Our diagnosis therefore was of a congenital extrahepatic portosystemic shunt manifesting with adult-onset encephalopathy.

In 1982, the first case of adult-onset encephalopathy associated with a noncirrhotic, extrahepatic, portosystemic shunt was documented in a 67-year-old woman with a vascular connection between her SMV and IVC [7]. Since then five similar reports have been contributed to the English language literature [8–11] and ten cases have been described in Japanese language journals.

The true incidence of congenital portosystemic shunts presenting in adulthood remains obscure, likely due to a combination of underdetection [12] and overreporting. For example, a nationwide Japanese survey of 120 hepatologists and pediatricians performed in 1999 revealed twenty-three cases of previously unreported adult-onset encephalopathy in patients in whom congenital extrahepatic portosystemic shunts had been diagnosed in childhood [13]. On the other hand, some case series fail to consider reasonable noncongenital shunt etiologies such as in five reported instances of isolated left gastric to left renal varices observed in patients who had undergone distal gastrectomy. In such instances, shunts may reasonably reflect postsurgical flow dynamics rather than preexisting congenital abnormalities [8].

The reason some congenital shunts remain clinically silent until adulthood is unclear. It has been suggested that the central nervous system becomes increasingly sensitive to hyperammonemia with age. Repeated spikes in serum ammonia due to high protein meals may serve as sensitizing triggers [8]. In the present case, the symptomatic threshold may have been crossed as the shunt grew in size: as blood flow is preferentially diverted from the IMV into the lower pressure renal vein, a perpetuating cycle of increasing flow volume and shunt hypertrophy would be expected to develop, although our ability to observe these dynamics is limited to the ten years for which cross abdominal sectional images of our patient are available. It is of note that all seventeen cases (including the present case) of adult encephalopathy from congenital extrahepatic shunts have been described in women between the ages of 35 to 76 years, which may suggest a role for vascular and hormonal dynamics of pregnancy contributing to the adult manifestation of congenital extrahepatic shunts.

In all but two of the cases previously reported, shunt ligation was achieved surgically. One patient was treated conservatively with lactulose and a low protein diet [11] and a second patient underwent endovascular coil embolization [10]. Endovascular coil embolization was described as an alternative to surgical ligation of cirrhotic portosystemic shunts in 1987 [14] and the technique has demonstrated consistent efficacy with relatively few risks [15]. Successful endovascular closure of congenital shunts has also been reported in five children between the ages of 3 to 14 years [16–18]. The Amplatzer II vascular plug occupies a variable length of vessel, is repositionable, and has active detachment making it useful as a versatile large embolic device for our procedure.

In creating an immediate diversion of flow into the portal venous system, the risk of iatrogenic portal hypertension must be considered. In this case, sonographic and angiographic evaluation confirmed slow portal venous inflow with no outflow obstruction. In the setting of known hepatic venous outflow compromise, a graduated, sequential technique for endovascular shunt closure has been described [19]. In summary, our case represents a rare diagnosis with devastating physiologic and psychological consequences, treated successfully via minimally invasive endovascular approach.
